# Supplementary material for: Are carbon emissions trading and green financial instruments synergistic? -Comprehensive quantitative research based on content analysis
Source: PLoS One. 2024 Mar 7;19(3):e0298601. doi: 10.1371/journal.pone.0298601 (PMC10919601; doi:10.1371/journal.pone.0298601)
Supplement: S1 Data — (ZIP) [file pone.0298601.s001.zip › Data/地级市Z_F工作报告.zip!/╔╜╬≈/╦╖╓▌╩╨/└┤╫╘╣1⁄2╓┌║┼í╛┬φ┐╦╩2╛▌═°í┐_2016.docx]

各位代表：

　　现在，我代表市人民政府向大会报告工作，请予审议，并请市政协委员和其他列席人员提出意见。

　　一、2015 年及“十二五”时期工作回顾

　　过去一年，面对经济下行压力持续加大的严峻形势，在市委的坚强领导下，我们认真贯彻落实党中央国务院稳增长促改革调结构惠民生政策措施和省委省政府决策部署，紧扣“两大任务”工作主题，积极应对挑战，锐意攻坚克难，重要工作取得新突破新亮点新进展。

　　2015年，全市经济总体运行缓中趋稳。地区生产总值完成901亿元，同比下降2.3%，降幅比上半年收窄2.3个百分点；固定资产投资完成937亿元，增长14.9%，高于全省增幅；社会消费品零售总额完成270.1亿元，增长4.6%；公共财政收入完成54.3亿元，下降37.3%，降幅比上半年收窄3.7个百分点；居民人均可支配收入达到18983元，增长7.9%，城乡居民收入继续跑赢经济增速。物价总水平处于低位，就业形势基本稳定，产业结构发生积极变化，经济增长质量继续改善，新技术、新业态、新模式“三新经济”加速孕育，经济社会发展在克服困难中奋力前行。

　　全力以赴稳增长，项目建设成效显著。千方百计稳定工业运行。认真落实省政府减轻企业负担“60条”，采取金融支持、财政扶持等一系列政策措施，落实小微企业减免税费等优惠政策，为企业减负43亿元，企业成本明显下降。大力开展“冬季行动”，“一企一策”精准帮扶企业，有效缓解了企业困难，工业经济降幅逐步收窄，年末连续四个月当月实现正增长。狠抓投资增长和项目建设。扎实开展“项目提质增效年”活动，坚持“六位一体”，采取领导包项目、现场解难题、每月抓调度、全市一盘棋、项目大起底等方式，目标导向倒逼，层层传导压力，实现固定资产投资增速逐月提升，省市重点工程完成投资770.9亿元，一批打基础、利长远的重大项目落地建设。平朔木瓜界2×66万千瓦、平朔安太堡2×35万千瓦、同煤朔南2×35万千瓦3个低热值煤发电项目和中电国际神头发电公司2×100万千瓦火电项目开工建设，开工火电项目全省最多。蒙西至天津南、晋北至江苏两条特高压输电线路顺利开工，为建设综合能源基地拓宽了发展空间。右玉至平鲁高速公路开工，这是去年全省高速公路建设投资最多的项目。特别是经过努力推动，大西高铁朔州段前期工作正式启动，朔州机场选址通过专家评审，全市人民长期关注的两个重大项目取得突破性进展。努力促进消费。积极开展各类促进消费活动，推进城市综合体建设，完善城乡商贸流通服务体系，美都汇购物广场、怀仁汽车文化城等项目建成运营，全社会消费保持稳定增长。

　　深入推进产业结构调整，转型升级迈出坚实步伐。大力发展特色现代农业。加强农田水利基本建设，农田实灌面积达到210万亩。在旱灾严重的情况下，粮食总产量仍达到22亿斤，为历史上第三个高产年。我市被农业部列为全国唯一的草牧业发展试验试点市，试点工作取得积极成效。奶牛存栏量、肉羊出栏量稳定增加。全市农产品加工龙头企业销售收入达到180亿元，比上年增长12.5%。改造提升传统产业。加快推进现代化矿井建设，标准化矿井达到45座。全年生产原煤1.82亿吨。大力发展低热值煤发电、风力发电、光伏发电，新增电力装机容量166.75万千瓦。全年发电267.5亿度，其中风力发电23.3亿度，增长30.7%。加快培育新兴产业和服务业。铺开装备制造、食品、医药等新兴产业项目126个，完成投资106亿元。非煤电产业投资占工业投资比重达到34.5%，比上年提升3.1个百分点。服务业保持稳定增长，占地区生产总值比重达到48.7%。全市旅游总收入达到133.56亿元，增长22.2%。

　　加强基础设施建设，城乡环境面貌得到改善。推进中心城市建设。继续实施七里河综合治理、朔州老城改造，新建和改造城市道路51公里、各类市政管网388公里，新增城市集中供热面积492万平方米，完成市污水处理厂提标改造，基本建成市第二污水处理厂。城市总体规划获得省政府批复，市区两级规划管理实现统一。改善城乡人居环境。扎实推进设施提升、城市安居、城中村改造和环境提质城市人居环境改善“四大工程”，全年完成投资154.39亿元；其中新开工各类保障性住房13613套，基本建成20522套，完成投资35.74亿元。继续推进完善提质、农民安居、环境整治、宜居示范农村人居环境改善“四大工程”，认真办好农村“五件实事”，完成投资12.6亿元。加强生态建设。积极开展植树造林，完成营造林22万亩，超省考核任务30%。强化节能减排，狠抓大气污染防治，万元地区生产总值综合能耗下降5.35%，万元工业增加值用水量下降3%，主要污染物减排任务全部完成，空气质量得到好转。

　　全面推进改革开放，发展动力显著增强。扎实推进重要领域和关键环节改革。农村土地承包经营权确权登记颁证全面展开，煤炭管理体制改革迈出重大步伐，财税体制改革深入推进，转型综改试验区建设取得突破性进展。加大科技创新力度。搭建大众创业、万众创新平台，利用人防疏散基地建设市科技创新园区及大学生创业基地。设立企业技术研发创新项目专项资金，支持企业技术研发，新增企业技术中心6家、高新技术企业2家。与北京大学合作共建的固废资源化研究中心，被认证为国家大宗工业固废及资源化产品质量监督检验中心。推动金融振兴。全市社会融资总量达到119.5亿元，增长11.1%。引进华夏银行设立分支机构，完成怀仁县、应县村镇银行筹建工作，成立创业就业小额贷款担保中心，新增一家注册资本金1亿元的民营融资性担保公司。朔州煤电宏力再生工业公司在“新三板”上市。支持民营经济发展。出台发展民营经济实施意见，推进政府与社会资本合作，民间投资占全市固定资产投资比重达到40.9%。推进工商登记便利化，新增民营企业和个体工商户11089户，增长17.9%。扩大招商引资。主动对接京津冀、沿海地区和大型企业，全年签约招商引资项目119个，到位外来资金606.2亿元。

　　着力保障和改善民生，人民生活水平进一步提高。在财力紧张情况下，保障民生力度持续加大。全市财政在民生领域投入113.1亿元，占到公共财政预算支出的81.78%。积极促进创业就业，城镇新增就业23109人。实行机关事业单位养老保险制度改革，全面提高企业退休人员基本养老金、城乡居民基础养老金标准、城镇居民医保补助、新农合人均筹资标准、城乡低保标准、农村五保户供养标准。加强义务教育学校标准化建设。支持中北大学办好朔州校区，在校生达到3200名。推开县级公立医院改革，全面实施城乡居民大病保险制度，群众看病贵问题得到有效缓解。加强老年人健康服务，为38249名60岁至64岁老年人提供了免费健康体检。实施文化惠民工程，开展送戏下乡、周末大舞台等活动，丰富群众文化生活。发展体育事业，竞技体育水平得到提高，在第十五届省运会2015年度比赛中取得3金3铜的好成绩。加大扶贫攻坚力度，50个贫困村、23387名贫困人口实现脱贫。向农村居民发放供暖补贴1.33亿元。

　　认真办好十件实事。深入推进国家公共文化服务体系示范区建设，县区图书馆、文化馆全部达标；政府通过购买服务方式，向高校毕业生提供1452个就业岗位；市级财政投入600万元，解决了35个村、1.35万人饮水困难；各级财政投入8.9亿元，统筹推进采煤沉陷区治理、地质灾害治理、农村危房改造、扶贫易地搬迁，安置6716户农民，易地搬迁1411人；开展全民参保登记，入户调查156.23万人；新改扩建农村五保户集中供养服务机构6所，新增床位394张；全面实施城乡清洁工程，创建达标村345个；市一中高中新校区、朔州大医院前期工作积极推进；乡镇卫生院医务人员得到充实。广大人民群众享受到了更多改革发展成果，有了更多获得感。

　　高度重视安全生产，扎实开展安全生产大检查，加强隐患排查治理，各类安全生产事故死亡人数同比下降27.14%。认真做好信访工作，严厉打击各类违法犯罪，社会保持和谐稳定。加强国防后备力量建设，积极开展双拥工作，我市被评为省级双拥模范城。与此同时，发展妇女儿童、老龄和残疾人事业，民族、宗教、外事、侨务、地震、气象、档案、史志等各项工作都取得新成绩。

　　加强政府自身建设，发展环境不断优化。全面推行“六权治本”，制定实施政府自身建设三年规划和年度行动计划。顺利完成新一轮政府机构改革。积极推进行政审批制度改革，承接、下放、取消、调整行政审批事项77项，保留204项。公布运行市级权力清单和责任清单。通过简政放权，进一步释放了市场活力，降低了企业成本，推动了创业热潮。加强党风廉政建设和反腐败斗争，认真执行中央八项规定精神和国务院“约法三章”，狠刹“四风”，“三公”经费大幅减少，一些违法违纪领导干部受到严肃查处。公务用车改革有序推进，涉改公车全部封存。自觉接受人大和政协监督，支持各民主党派、工商联、无党派人士参政议政。市六届人大一次会议以来，共办理人大代表建议和政协委员提案266件。加强作风建设，大力弘扬右玉精神，认真开展“三严三实”专题教育和学习讨论落实活动，干部作风不断改进，政务服务质量进一步提升。

　　各位代表，2015年是“十二五”收官之年。“十二五”时期，面对复杂严峻的经济形势和艰巨繁重的改革发展稳定任务，我们认真贯彻落实党的十八大、十八届三中、四中、五中全会精神和习近平总书记系列重要讲话精神，按照省委“五句话”总要求，加快推进“六大发展”，全面实施“六权治本”，统筹做好煤与非煤两篇文章，大力推动“三个突破”，紧扣“两大任务”工作主题，紧抓循环经济、新兴产业、特色农业、生态建设、城乡统筹“五大重点”，全力打造全国综合能源基地、全国工业固废综合利用示范基地、全国日用陶瓷生产基地、全国生态畜牧养殖基地、全省特色农产品加工基地“五个基地”，着力提升煤炭产业循环率、新兴产业占比率、绿化覆盖率、城镇化率和城乡居民收入水平，坚持稳中求进总基调，积极适应经济发展新常态，加快建设美丽朔州和“塞上明珠”，全市经济社会发展取得新成就，“十二五”规划确定的主要目标任务基本完成，为全面建成小康社会打下坚实基础。一是综合经济实力显著增强。地区生产总值五年累计完成4793.1亿元，是“十一五”的2.16倍，年均增长7.3%；固定资产投资累计完成3612.2亿元，是“十一五”的3.15倍，年均增长21.3%；城镇居民人均可支配收入达到27500元，是“十一五”末的1.57倍；农村常住居民人均可支配收入达到10816元，是“十一五”末的1.83倍。二是转型升级取得重要进展。“五大基地”建设成效显著。大力推进新型综合能源基地建设，全市煤炭生产能力、洗选能力、运输能力均达到2亿吨以上，位居全省第一；电力运营装机容量达到953.35万千瓦，千万千瓦级电力基地基本建成；风电、光伏发电等新能源电力规模达到296.35万千瓦，其中风电253.95万千瓦，居全省第一。大力推进工业固废综合利用示范基地建设，建成145家工业固废综合利用企业，年消化工业固废3000万吨以上，综合利用率达到63%以上，产业化水平走在全国前列。我市被列为全国工业固废综合利用示范基地、全国工业绿色转型试点城市和资源综合利用“双百”示范基地。连续三年举办亚洲粉煤灰及脱硫石膏处理与利用技术国际交流大会。大力推进日用陶瓷生产基地建设，日用瓷年生产能力达到18.5亿件。大力推进生态畜牧养殖基地建设，实施雁门关生态畜牧经济区领头雁工程和核心区建设，启动国家草牧业试验试点市建设，六县区全部进入全省30个牛羊产业重点县行列，奶牛存栏量、鲜奶产量、肉羊出栏量、人均畜产品占有量、农民人均畜牧业纯收入五项指标连续多年保持全省第一。大力推进特色农产品加工基地建设，农业产业化经营组织达到1218家，全省产业化重点龙头企业达到28家，销售收入超亿元企业达到25家。三是城乡环境质量全面提升。深入开展“五城联创”，加强中心城市建设，实施城市人居环境改善工程，推进“一山两河一湖”生态体系建设。成功创建国家园林城市，城市建成区绿化覆盖率达到42.56%。完成新一轮农村“五个全覆盖”工程，持续办好农村“五件实事”，实施农村人居环境改善工程，村容村貌明显改善。公路通车里程突破1万公里，实现县县通高速、村村通公路。全市城镇化率达到53.2%，比“十一五”末提高6.9个百分点。平均每年营造林超过30万亩，森林覆盖率预估达到24%。四是社会发展成就斐然。持续推进国家级创业型城市建设，五年新增城镇就业11.83万人。逐年提高城镇职工退休养老金、城乡居民低保、最低工资等社会保障标准，覆盖城乡的社会保障体系基本建立。推进义务教育学校标准化建设，六县区全部通过省级评估。建成朔州师范专科学校、朔州职业技术学院、中北大学朔州校区，高等教育迈上新台阶。深化医药卫生体制改革，基层医疗卫生机构全面实行基本药物制度。推进国家公共文化服务体系示范区建设，覆盖城乡的公共文化设施网络初步建成。实施“双稳定”农民增收工程，农村居民人均可支配收入突破万元大关，高于全省平均水平。大力实施脱贫攻坚，10.7万贫困人口实现脱贫。狠抓安全生产，安全生产事故死亡人数逐年下降。加强法治朔州建设，我市被评为全国社会管理综合治理先进市、“六五”普法中期全国先进市，连续11年被表彰为全省社会治安综合治理先进市。五是改革开放不断深化。以转型综改试验区建设为统领，深入推进行政审批制度改革、商事制度改革、煤炭管理体制改革、农村改革等重点领域改革，实施科技创新驱动战略，推动金融振兴，支持民营经济发展，不断加大招商引资力度，发展的动力和活力持续增强。

　　各位代表，“十二五”时期经济社会发展取得的成绩来之不易。这是省委、省政府和市委正确领导的结果，是市人大、市政协有效监督、全力支持的结果，是全市人民齐心协力、共同奋斗的结果。在这里，我代表市人民政府，向全市人民和所有关心支持朔州发展的各界人士，表示衷心的感谢和崇高的敬意!

　　在肯定成绩的同时，我们也要清醒地看到，当前，我市发展面临多重困难和严峻挑战，长期积累的结构性矛盾开始释放。煤电支柱产业持续低迷，投资增长乏力，大项目好项目储备不多，个别主要经济指标出现多年未有的负增长，稳增长压力巨大；新兴产业发展缓慢，科技创新引领和人才支撑作用不足，转型升级十分艰难；基础设施建设滞后，公共服务水平较低，脱贫攻坚时间紧迫，民生改善任务繁重；财政收入大幅减少，刚性支出不断增加，收支平衡难度很大；安全生产形势不容乐观，信访问题突出，社会治理还有不少薄弱环节，维护社会和谐稳定面临考验；政府职能转变还不到位，少数干部不担当、不作为、工作落实不力，作风建设须进一步加强。特别要注意的是，伴随去产能、去库存、去杠杆，我市经济内生向下调整的压力更大，企业资金链、房地产市场、社会就业、地方财力将承受更大冲击。我们一定要增强危机意识、创新意识、进取意识、担当意识，夙夜在公，一心为民，尽心竭力做好政府工作，努力推动经济社会平稳健康发展。

　　二、“十三五”时期经济社会发展的指导思想和目标任务

　　根据《中共朔州市委关于制定国民经济和社会发展第十三个五年规划的建议》，市政府编制了《朔州市国民经济和社会发展第十三个五年规划纲要（草案）》，提交大会审议。

　　“十三五”时期经济社会发展的指导思想是：高举中国特色社会主义伟大旗帜,全面贯彻党的十八大和十八届三中、四中、五中全会精神，以马克思列宁主义、毛泽东思想、邓小平理论、“三个代表”重要思想、科学发展观为指导，深入贯彻习近平总书记系列重要讲话精神，遵循“五位一体”总体布局和“四个全面”战略布局，坚持发展是第一要务，牢固树立并切实贯彻创新、协调、绿色、开放、共享的发展理念，按照省委“五句话”总要求和“六大发展”战略部署，主动适应经济发展新常态，紧扣优化经济结构和提升发展质量“两大任务”工作主题，进一步突出循环经济、新兴产业、特色农业、生态建设、城乡统筹“五个重点”，打造全国综合能源示范基地、工业固废综合利用示范基地、日用陶瓷生产基地、生态畜牧养殖基地和全省特色农产品加工基地“五大基地”，统筹推进经济建设、政治建设、文化建设、社会建设、生态文明建设，努力建设美丽朔州和“塞上明珠”，奋力夺取全面建成小康社会决胜阶段伟大胜利。

　　主要目标是：发展质量明显提升。传统产业竞争力增强，新兴接替产业规模壮大，服务业比重提高，农业现代化迈上新台阶，“五大基地”建设取得重大突破，煤炭产业循环率、新兴产业占比率、绿化覆盖率、城镇化率实现既定目标。到2020年，全市地区生产总值和城乡居民人均可支配收入比2010年翻一番，我市与全国全省同步全面建成小康社会。贫困人口整体脱贫。“十三五”时期每年减少40个贫困村、2.1万贫困人口，现行标准下的8.3万农村贫困人口实现稳定脱贫，贫困县全部摘帽。生态建设成效显著。能耗和水资源消耗、建设用地、碳排放总量得到有效控制，主要污染物减排完成省定任务，大气、水、土壤污染治理取得新成效。森林、草地覆盖率进一步提高。民生保障水平普遍提高。就业水平比较充分，公共服务体系更加健全，基本公共服务均等化水平提高。城乡居民人均可支配收入增速不低于全市地区生产总值增速，农村居民收入增速快于城镇居民收入增速。安全生产向稳定好转坚实迈进。文化建设呈现新局面。文化发展主要指标、文化事业整体水平、文化产业综合实力明显提升，公共文化服务体系基本建成，文化产业发展格局基本建立，公民素质和社会文明程度显著提高。改革开放进一步深化。资源型经济转型综合配套改革试验区建设取得重大进展，重点领域和关键环节改革取得决定性成果，对外开放的广度和深度不断拓展。民主法治建设扎实推进。法治政府基本建成，政府公信力和行政效率进一步提高。民主法治更加健全，社会更加和谐稳定。

　　主要任务是：

　　（一）坚持创新发展，着力提高经济发展质量和效益。坚持把创新摆在发展全局的核心位置，深入实施创新驱动发展战略。着力加强供给侧结构性改革，发挥有效投资对经济增长的关键作用，发挥消费对经济增长的基础性作用。大力推动大众创业、万众创新。做好煤与非煤两篇文章，实施“革命兴煤”，推进煤炭“六型转变”，做优做强能源产业，培育发展新兴接替产业，提升服务业发展水平，加快发展特色现代农业。深入推进转型综改试验区建设，深化重点领域和关键环节改革，全力推进科技创新、金融振兴、民营经济发展“三个突破”。实施人才强市战略。

　　（二）坚持协调发展，着力形成均衡发展格局。按照中心城市、大县城、小城镇、新农村“四位一体”发展思路，坚持工业反哺农业、城市支持农村，加快构建城乡一体的发展机制，推进新型城镇化。力争到2020年，常住人口城镇化率达到60%，户籍人口城镇化率达到40%左右。深化“五城联创”，提高城市建设管理水平。深入开展社会主义核心价值观教育实践活动、思想道德建设活动和群众性精神文明创建活动，大力弘扬右玉精神，繁荣文化事业，发展文化产业，促进物质文明和精神文明协调发展。推动军民融合向全要素、多领域、高效益深度发展。

　　（三）坚持绿色发展，着力建设生态文明美丽朔州。实施主体功能区战略，严守生态安全红线。推行低碳循环生产生活方式，促进资源节约高效利用。构筑绿色生态屏障，实施大规模国土绿化行动，加强水生态建设。实行最严格的环境保护制度，实施大气、水、土壤污染防治行动计划，加强矿山生态环境恢复治理，加大城乡环境整治力度。严格落实生态环境保护法律法规，打击各类环境违法行为。

　　（四）坚持开放发展，着力拓展经济发展空间。紧紧抓住“一带一路”倡议机遇，主动融入京津冀一体化和环渤海经济圈，积极对接长三角、珠三角，深化区域合作，加强开发区和园区等各类开放平台建设，承接产业转移，提高招商引资质量和水平。扩大外贸规模，提升经济外向度。

　　（五）坚持共享发展，着力创造人民幸福美好生活。始终坚持把保障和改善民生作为一切工作的出发点和落脚点，全面提升教育、医疗、就业、社会保障、文化等民生领域公共服务水平，提高城乡居民收入水平，坚决打赢脱贫攻坚战，促进人口均衡发展，使全市人民在共建共享发展中有更多获得感。

　　（六）坚持廉洁发展和安全发展，着力营造良好发展环境。严格落实“两个责任”，深入推进“六权治本”，持续狠刹“四风”，营造廉洁发展环境。全面加强安全生产，夯实安全发展基础。加强和创新社会治理，强化社会治安综合治理，健全公共安全保障体系，维护社会和谐稳定。

　　各位代表，做好“十三五”时期经济社会发展工作，实现全面建成小康社会目标，必须牢牢抓住发展第一要务不放松，大力推进结构性改革，加快发展新经济。“十三五”规划纲要（草案）承载着全市人民对美好生活的殷切向往，《纲要（草案）》经本次会议审议通过后，我们将全力抓好落实。我们坚信，通过全市人民的共同努力，我们一定能够夺取全面建成小康社会的伟大胜利！三、2016年工作安排

　　2016年，是全面建成小康社会决胜阶段的开局之年。我们要积极适应经济发展新常态，坚持稳中求进总基调，着力加强供给侧结构性改革，统筹稳增长、调结构、惠民生、防风险，抓好去产能、去库存、去杠杆、降成本、补短板，努力实现“十三五”良好开局。

　　今年经济社会发展的主要预期指标是：地区生产总值增长6.5%左右，固定资产投资增长15%，社会消费品零售总额增长6%左右，公共财政预算收入下降15%，城镇常住居民人均可支配收入增长6.5%左右，农村常住居民人均可支配收入增长6.5%以上。约束性指标完成省下达任务。

　　做好今年工作，总的要求是确保经济平稳运行、稳中向好，生产总值、投资、物价、就业、收入等重要经济指标努力保持在合理区间。

　　重点做好九个方面工作。

　　（一）推进供给侧结构性改革，加快产业转型升级

　　围绕做好煤与非煤两篇文章，改造提升传统产业，加快发展新兴产业，促进产业结构不断优化。继续简政放权、减税清费，让企业休养生息，让“双创”结出更多成果，推动全市经济向更高层次奋力跃升。

　　做优煤电产业。一要促进煤炭行业脱困转型。继续推进煤炭资源整合、煤矿兼并重组，依法淘汰落后产能，有序退出过剩产能，严控增量，优化存量。精心组织煤炭企业合理生产，积极搭建平台，推动产运销用各方面密切协同。支持大企业收购地方煤矿煤炭，鼓励本地企业使用本地煤，稳定煤炭销量。二要促进能源产业清洁高效发展。大力推进煤电一体、煤化一体、煤电铝一体，提高煤炭清洁高效利用水平，打造煤炭产业升级版。着力推进煤转电，抓紧建设同煤朔南等四个低热值煤发电项目和中电国际神头发电公司“上大压小”二期项目，争取年内有新项目建成。加快建设蒙西至天津南、晋北至江苏两条特高压输电线路，提高电力输出能力。推进煤化一体化发展，引进阳煤集团、北京神雾集团等大企业利用新技术建设煤炭分级分质利用项目，推动建设晋北现代煤化工基地。推进煤电铝一体化发展，抓住电力体制改革机遇，拓展现代高载能产业。支持朔州铝硅公司与漳泽电厂合作，承接电解铝产能转移。积极发展新能源产业，加快建设装机规模84.1万千瓦的风电和光伏发电在建项目，推动装机规模92.4万千瓦的14个风电和光伏发电前期项目尽早获批开工，引进唐山海泰新能科技公司太阳能多晶硅项目落户富甲园区。

　　做大非煤产业。推动陶瓷产业升级，鼓励陶瓷企业兼并重组，培育自主创新能力强的行业龙头。承接山东、广东等沿海地区陶瓷产业转移，引进山东临沂华伦建陶公司高档地砖项目落户应县。设立陶瓷产品创新奖，支持陶瓷企业研发新产品。大力发展装备制造业，推动平朔公司与徐工集团合作建设矿山工程机械项目。发展电动汽车产业，在注册登记、政府采购等方面实行倾斜政策，规划建设充电桩等基础设施。加快发展食品医药产业，壮大右玉梁威工业园和怀仁医药园区产业规模。积极发展煤基新材料和节能环保产业。

　　加快发展现代服务业。突出发展文化旅游业。培育文化旅游业龙头企业，组建文化旅游产业投资公司。进一步完善旅游景区基础设施，深度开发旅游产品。支持右玉县创建国家全域旅游示范区，引进投资主体开发大广武景区，推进应县南山生态旅游项目。着力发展现代物流业，抓好雁门关农产品物流园区等项目建设。提升商贸服务业。加强商业网点体系建设，抓好北京电子城朔州数码港、普国边塞国际商城等重点项目建设，优化消费环境，促进消费增长。积极发展电子商务。大力发展健康养老业。市里和各县区都要建设好一个养老示范项目。落实财政补贴政策，鼓励社会资本兴办健康养老项目。

　　精准帮扶企业。认真落实国家和省扶持政策，降低企业综合成本，减轻企业负担。加大帮扶企业力度，帮助困难企业妥善解决职工就业、工资、社保等问题。推动企业加强管理，挖潜增效。

　　（二）加大投资和项目建设力度，增强持续增长动力

　　应对经济下行压力，必须充分发挥有效投资对经济增长的关键作用，既要规模又要结构，既要速度更要效益。

　　加强重点领域投资和项目建设。我市基础设施和民生领域有许多短板，产业亟须改造升级，有效投资仍有很大空间。着力推进基础设施、产业转型、生态建设、民生改善四个方面八大领域投资和标志性工程建设,铁路项目投资13.6亿元，公路项目投资20亿元，电力项目投资235亿元，现代煤化工项目投资34.5亿元，新兴产业项目投资87.6亿元，生态环境治理项目投资56.8亿元，城乡人居环境改善项目投资100亿元，社会事业项目投资19.5亿元。八大领域投资占到全市计划总投资的一半以上。突出抓好大西高铁朔州段、环城高速东北环、市区至高铁站快速通道、雁阳公路、朔州机场、中海油蒙西煤制天然气外输管道工程等重大标志性工程，力争早日开工建设。

　　加大项目和政策争取力度。加强与国家、省相关部门对接，努力争取一批公路、水利、电力等领域的大项目。加快推进项目前期工作，积极组织申报条件成熟的项目，争取国家专项建设基金。各县区要策划储备一批能够有效拉动投资的大项目好项目。

　　深化投融资体制改革。强化政府投资引导作用，发挥企业投资主体作用，激发民间投资积极性，扩大债券融资规模，推广特许经营、股权合作、政府购买服务等政府和社会资本合作模式，通过多渠道多形式筹措建设资金。

　　完善推进项目建设工作机制。继续坚持“六位一体”，强化市县领导包项目责任制、挂牌督办制、月调度月考核月排名等行之有效的制度。健全重大项目联席会议机制，协调解决手续办理、征地拆迁、环评、阻工等实际问题，确保项目及时落地、顺利推进。

　　（三）加强“三农”工作，促进农民持续增收

　　始终把“三农”工作作为重中之重，抓住国家草牧业发展试验试点市建设机遇，深入实施“双稳定”农民增收工程，加快转变农业发展方式，推动雁门关生态畜牧经济区核心区建设迈上新台阶。

　　大力发展特色现代农业。扎实推进草牧业发展试验试点市建设，全面实施草牧业“双百双十”工程，今年完成人工种草57万亩，改良天然草场30万亩。每个县区要建设好一个百亩以上的牧草种植试验示范点。统筹发展草牧业与养殖业，改造提升38个奶牛养殖园区、40个肉羊养殖园区。调整优化种植结构，实施高产创建工程，建设100万亩玉米地膜覆盖高产田、100万亩优质杂粮生产基地、100万亩高效园艺作物基地和50万亩优质马铃薯生产基地。加强农田水利建设，提高农业综合生产能力。

　　加快构建农业经营新体系。积极发展家庭农场、专业大户、农民合作社、农业产业化龙头企业，做大做优乳品、肉制品、酒类、小杂粮、蔬菜等特色农产品加工业，提高农业产业化经营水平。年内认定100名新型职业农民、100个家庭示范农场、100个农民合作示范社、10家农业示范企业。全年农产品加工销售收入实现195亿元。推动农业一二三产业融合发展。大力发展休闲度假、旅游观光、农耕体验、创意农业等农村经济新业态，培育农民增收新的增长点。加强农业社会化服务体系建设，推进供销社综合改革。加大农业科技创新支持力度，发展农业科研机构，与省农科院合作建设生态畜牧研究院。加快推进主要农作物全程机械化，新增农机总动力4万千瓦。

　　深化农村改革。深入推进农村改革试验区建设，基本完成农村土地承包经营权确权登记颁证工作。建设农村产权交易市场，促进土地有序流转。推进农村集体产权制度改革，发展农村集体经济，每个县区都要抓好一个试点村。稳步推进水权制度和小型水利工程管理体制改革，启动河湖划界确权工作。加强农村金融服务体系建设，开展农村土地承包经营权、林权、农业生产设施抵押质押试点，完善农业保险制度。

　　打好脱贫攻坚战。坚持精准扶贫、精准脱贫，扎实开展建档立卡“回头看”，按照扶持对象、项目安排、资金使用、措施到户、因村派人、脱贫成效“六个精准”要求，创新脱贫攻坚机制，组织实施发展生产、易地搬迁、生态补偿、发展教育、社会保障和干部驻村帮扶“六个一批”工程。严格落实脱贫攻坚领导责任制，层层签订责任书，立下军令状。市委、市政府负责上下衔接，域内协调，督促检查。县区党委、政府承担主体责任，县委书记和县长是第一责任人，抓好进度安排、项目落地、资金使用、人力调配等关键环节。特别是右玉、平鲁、山阴三县区要做好统筹规划，用好政策资金，坚决不落步子。落实行业部门扶贫责任，加大财政扶贫资金投入，引导社会力量参与。建立健全脱贫攻坚考核机制，确保按时保质保量完成各项任务。今年要实现2.1万贫困人口脱贫、40个贫困村摘帽。

　　（四）做好城市工作，积极推进新型城镇化

　　紧紧围绕建设自然生态精致宜居现代化城市目标，坚持科学布局、规划先行、建设为基、管理为要，努力走出一条富有活力、和谐宜居、各具特色的现代城市发展之路。

　　强化城市规划工作。着力提升城市规划水平，打好城市长远发展的坚实基础。增强规划的科学性，创新城市规划理念，引领城市发展。增强规划的系统性，推进“多规合一”，实现城市总体规划与土地利用、环境保护、产业发展等规划有效衔接。维护规划的权威性，严格按照城市总体规划和控制性详细规划建设管理城市，坚决杜绝随意修改规划，严肃查处违反规划的建设行为。

　　加快完善中心城市基础设施。重点抓好十大城建工程。一是加快推进七里河市区段生态治理工程，基本完成建设任务。二是启动太平窑水库治理，建成城市周边湿地公园。三是开工建设“三路四桥”，打通顺义路、振华街、安泰街，配套建设城市地下综合管廊，完善城市路网。四是加快建设恢河大桥，解决市区南北交通瓶颈问题。五是大力推进七里河沿线城中村改造，让广大城中村群众有一个良好的生活环境。六是完善朔州老城改造工程，建成特色文化旅游景区。七是开工建设四水厂和西南片应急水源，提高城市供水能力。八是建设垃圾处理第二填埋区渗沥液处理站和垃圾中转站，完善垃圾处理设施。九是投产运营市第二污水处理厂，提高污水达标排放水平。十是建成公交调度中心，投放新能源公交车，扩充城市公交运力，形成较为完善的城市公共交通服务体系。

　　统筹改善城乡人居环境。进一步实施城市人居环境改善工程，提高城镇宜居宜业水平。重点抓好城镇保障性安居工程建设，今年全市开工6000套，其中城中村改造1715套。统筹推进城镇保障性安居工程和房地产市场平稳健康发展，加大棚改货币化安置力度，积极发展住房租赁市场，打通商品房、保障房、安置房转换通道，鼓励农民和农民工进城购房，扩大有效需求，化解房地产库存。继续实施农村人居环境改善工程，加强农村水电路气等基础设施建设，新建改造饮水工程83处，新改扩建农村幼儿园9所，建设40个农村社区老年人日间照料中心，实施采煤沉陷区治理搬迁、农村困难家庭危房改造、易地扶贫搬迁、农村住房抗震改建共17317户，启动65个村地质灾害治理搬迁，创建10个美丽宜居示范村和580个清洁达标村。

　　健全城镇化推进机制。深入实施大县城战略，加强特色小城镇建设，容纳更多的农民工就近就业创业，促进就地城镇化。推动每个县区打造一两个链条比较长、带动能力强的特色产业，加快壮大县域经济，提高县域城镇人口承载能力。加快户籍制度改革，推行居住证制度，促进农业转移人口市民化。完善城镇基本公共服务，努力实现常住人口全覆盖。维护进城落户农民土地承包权、宅基地使用权、集体收益分配权，支持其依法自愿有偿转让。

　　全面加强城市管理。积极推进城市管理体制改革，合理划分市区两级管理事权，推行综合执法。创新城市管理方式，推进智慧城市管理，建设城市管理数字化平台,提升城市精细化管理水平。深入推进“五城联创”，巩固国家园林城市创建成果，卫生城市、环保模范城市、双拥模范城市、文明城市创建工作要在已经获得省级称号的基础上，加快国家级城市创建步伐。完善城市应急体系，保障城市安全稳定运行。

　　（五）实施创新驱动，深化改革开放

　　改革才有活力，开放才有空间。我们要进一步解放思想、大胆探索，深入推进转型综改试验区建设，抓好重点领域改革，加快创新体制机制，激发更多发展动力。

　　着力推进创新驱动。强化企业科技创新地位，积极实施“互联网＋”行动计划，加快提高全市科技创新能力。今年重点抓好“五个一批”，建好一个平台。推动规上工业企业新建一批企业技术中心，力争年内全市企业技术中心突破100家；申报审批一批重点实验室（工程技术研究中心），年内省市级重点实验室达到10家以上，支持中煤集团建设煤炭提取稀有金属研发中心；实施一批重大科技攻关项目，推进开发区中美新能源技术研发公司洁净煤技术研发等项目，增强科技对经济社会发展的驱动作用；发展一批高新技术企业，全市高新技术企业达到20家；申报一批专利技术，年末有效发明专利超过150件；建好一个科技创新平台，把朔州市科技创新创业园打造成新技术新产品研发基地、中小微科技企业孵化基地、大众创业培训基地。同时，深化科技管理体制改革，提高科技项目和资金使用实效性。

　　加快金融改革发展。推动金融机构创新金融产品和融资模式，壮大社会融资总量，加大对地方经济支持力度。加强信用担保体系建设，整合财政扶持资金，设立中小企业信用保证基金。推进地方金融机构改革，加快完成朔州农村商业银行组建工作。加强金融体系建设，积极发展村镇银行，规范发展小额贷款公司，引进更多金融机构。积极培育上市公司。防范金融风险，打击非法集资，营造良好金融生态环境。

　　大力发展民营经济。落实各级政策，从项目、财税、金融、用地、人才等方面加大对民营经济的支持力度。设立专项发展基金，支持中小企业创业基地建设。深入开展“朔州企业家投资在朔州”活动，鼓励引导民营资本投资新兴产业和公共服务领域。帮助民营企业明晰产权，建立现代企业制度，成为合格市场主体。

　　不断提高开放水平。发挥资源优势，依托产业基础，提供优质服务，加大招商引资力度。依托山西品牌中华行、山西品牌丝路行，深化与“一带一路”沿线国家和地区交流合作。积极参与“民企入晋”招商引资系列活动，组织参加好省政府在南京、天津、重庆、济南、太原举办的五个招商对接会。发挥好开发区和园区作用，积极引进新兴产业项目。继续办好亚洲粉煤灰及脱硫石膏处理与利用技术国际交流大会，打造具有国际影响力的新技术新成果展示平台。

　　（六）着力保障改善民生，不断增进人民福祉

　　财政收入增长虽然放缓，但该给群众办的实事一件也不能少。坚决保障民生投入，加快完善基本公共服务体系，不断提高人民生活水平。

　　加快提高教育质量。积极发展学前教育，加大公立幼儿园建设力度。加强义务教育学校标准化建设，推动朔城区、山阴县、应县今年通过国家义务教育均衡发展评估认定。加强市区中小学校建设，缓解班容量大的问题。积极发展高中教育，推进市一中高中新校区建设。加快发展职业教育，加强特殊教育，支持办好高等教育。鼓励和规范发展民办教育。深化教育领域改革，优化教育资源配置。

　　提升医疗卫生保障服务水平。深入推进医药卫生体制改革，完善医疗卫生服务体系。深化县级公立医院改革，改善乡镇卫生院条件，推进朔州大医院建设。协调推进医疗、医药、医保联动改革。整合城乡居民基本医保制度，进一步提高财政补助标准和个人缴费标准，提高大病保险报销水平，扩大门诊慢性病病种，稳步推进跨省异地就医结算。全面实施一对夫妇可生育两个孩子政策，提升妇幼健康服务水平。加强食品药品综合治理，保障群众饮食用药安全。

　　着力扩大就业创业。深化国家级创业型城市创建活动，完善创业扶持政策，推动大众创业、万众创新。加强创业示范和实训基地建设，落实好创业担保贷款政策，完善公共就业服务体系。继续实行政府购买基层公共服务岗位等政策，促进高校毕业生就业。落实帮扶企业政策，稳定企业就业岗位。做好城镇失业人员、退役军人等群体的就业工作，托底帮扶就业困难人员。加强农民工职业技能培训。健全农民工工资支付保障机制。今年新增城镇就业岗位1.8万个，提供1000个政府购买基层公共服务岗位。

　　完善社会保障体系。稳步推进机关事业单位和企业基本养老保险制度并轨运行，继续提高退休人员基本养老金标准。提高城乡低保标准，推动农村低保标准与国家扶贫标准相衔接。落实经济困难的高龄与失能老人补贴以及百岁以上老年人补贴新标准。全面实施困难残疾人生活补贴和重度残疾人护理补贴制度。加强社保信息化建设，推进社保卡综合应用。做好社会救助，发展公益慈善事业。

　　加强文化建设。深入推进国家公共文化服务体系示范区创建工作，扎实完成各项任务，顺利通过国家验收。完善市县乡村四级公共文化服务场所建设，深化公益性文化事业单位内部改革。健全政府购买公共文化服务机制，开展好送戏下乡等文化惠民活动。做好文物和非物质文化遗产保护。加强科普工作，推进科技馆建设。发展体育事业，启动奥林匹克体育中心建设，倡导全民健身。深化群众性精神文明创建活动，提高市民素质和社会文明程度。

　　（七）加大环境治理力度，建设良好生态环境

　　治理污染、保护环境，事关人民群众健康和可持续发展，必须强力推进，努力实现经济发展与环境改善共赢。

　　推进低碳循环发展。实行能源和资源消耗、建设用地等总量和强度双控行动，促进节能、节水、节地、节材、节矿。深入开展重点行业能效对标活动，加强企业能耗在线监测。巩固提高煤矸石、粉煤灰等大宗工业固废综合利用水平，确保工业固废综合利用基地验收顺利通过。

　　深入开展大气污染防治行动。加快推进燃煤发电机组超低排放和节能改造，今年完成4家燃煤电厂5台机组改造任务，2017年全市所有燃煤电厂全部完成改造。进一步推进市区热电联产集中供热，取缔10蒸吨以下燃煤锅炉。强力实施市区城中村清洁燃煤改造，今年完成7个村1万余户改造任务。开展煤矸石自燃治理。加大建筑工地、道路扬尘污染治理力度。加强机动车污染防治。通过多管齐下、铁腕治污，实现空气质量明显好转。

　　扎实开展水污染防治行动。开展煤炭、陶瓷、食品、化工等行业的水污染防治设施改造。完善城镇污水处理设施。加强地下水水位、水质、水量监测，推动水资源合理利用。开展城乡集中饮用水水源地保护工作，保障居民饮水安全。

　　筑牢生态安全屏障。持续推进植树造林。今年完成17.43万亩营造林任务，重点抓好朔城区西山环城绿化九期、平鲁区龙雨山荒山造林、山阴县西山生态综合治理、怀仁县南家堡园林绿化、应县龙首山造林绿化和右玉县大南山荒山造林六大工程。结合草牧业试验试点工作，鼓励符合条件的地方开展新一轮退耕还林还草。加强生态水保工程建设，全面启动桑干河河流生态修复保护工程和七里河上游综合治理工程。推进矿山生态环境治理和地质灾害治理。

　　（八）毫不松懈抓好安全生产，保持社会和谐稳定

　　安全稳定责任重大，必须强化底线思维，警钟长鸣，常抓不懈，不断夯实经济社会发展基础。

　　牢固树立安全发展理念。坚持生命高于一切，牢记“三个决不能过高估计”的基本判断，坚决克服任何麻痹松懈思想，始终把安全生产放在突出位置。越是经济困难的时候，越要高度重视安全生产。

　　严格落实安全生产责任。按照“党政同责、一岗双责、失职追责”的要求，落实各级政府安全生产监管责任，切实做到管行业必须管安全、管业务必须管安全、管生产经营必须管安全。强化企业安全生产主体责任，切实加强现场管理，保障安全生产投入，不断改善安全生产基础条件。

　　狠抓隐患排查治理。市政府将今年确定为“安全隐患排查整治年”，深入开展煤矿、道路交通、油气输送管道、化工和危险化学品、冶金工贸、非煤矿山、水库、建筑工地、人员密集场所、消防等行业领域的安全大检查，集中排查治理重大隐患，坚决打击非法违法生产经营建设行为。

　　强化考核问责。严格落实安全生产目标责任考核“一票否决制”，继续实施企业重大隐患和事故挂牌督办制度。严肃查处事故，严格责任追究，促进安全生产形势持续稳定好转。

　　加强和创新社会治理。推进社区建设，提高基层社会管理和服务水平。支持工会、共青团、妇联等群团组织参与社会治理。切实保障妇女、儿童、残疾人权益，关爱老年人。深化平安朔州建设，落实社会治安综合治理领导责任制，完善立体化社会治安防控体系，扎实开展“打黑除恶”专项斗争深化年活动，严打暴力恐怖活动，有效防范和处置突发事件。深入推进法治朔州建设，深化司法体制改革，启动“七五”普法，强化法律援助。创新信访工作机制，有效化解矛盾纠纷。加强应急管理和防灾减灾能力建设。加强国防后备力量建设，支持军队深化改革，做好双拥工作。

　　（九）加强政府自身建设，提高施政能力和服务水平

　　新常态对政府自身建设提出了新的更高要求，要深入贯彻落实新发展理念，努力建设人民满意的法治政府、廉洁政府、创新政府和服务型政府。

　　深化“六权治本”。落实国家法治政府建设实施纲要，自觉运用法治思维和法治方式推动工作,法定职责必须为，法无授权不可为。健全重大行政决策机制，提高决策的科学化、民主化水平。完善“两清单”“两平台”运行制度，推动“六权治本”向乡村两级延伸。全面推行政务公开。自觉接受人大及其常委会监督，接受政协民主监督，重视社会舆论和社会监督，全面接受人民监督，让权力在阳光下运行。

　　加快职能转变。持续推进简政放权、放管结合、优化服务。深化行政审批制度改革，建立规范高效审批运行机制，提高行政服务效能。推进不动产统一登记工作。深化商事制度改革，提高注册登记便利化水平，为创业创新提供更好服务。

　　加强廉政建设。认真履行“一岗双责”，落实全面从严治党主体责任。严格执行中央八项规定精神和国务院“约法三章”，持续狠刹“四风”，厉行勤俭节约，反对铺张浪费。严控“三公”经费，有限财力要优先保障民生。加强行政监察和审计监督，严格监管公共资金、公共资源、国有资产。扎实开展重点领域和关键环节专项整治，坚决纠正侵害群众利益不正之风，坚定不移惩治腐败。

　　狠抓工作落实。深入践行“三严三实”，认真开展“两学一做”学习教育，牢固树立政治意识、大局意识、核心意识、看齐意识，恪尽职守、敢于担当，积极进取、善谋勇为，切实加强作风和能力建设。严格执行工作责任制，建立“马上就办、真抓实干”推进机制，市县两级要突出抓好市委提出的17项重点工作。加强常态化督查，紧盯不落实的事，问责不落实的人，严查不作为的官，确保各项任务落到实处、取得实效。

　　各位代表，我们面临的任务艰巨繁重，肩负的使命重大光荣。让我们更加紧密地团结在以习近平同志为总书记的党中央周围，在中共朔州市委的坚强领导下，凝心聚力，扎实苦干，实现“十三五”良好开局，为全面建成小康社会而努力奋斗！
